# Comparison of inequality in utilization of postnatal care services between Bangladesh and Pakistan: Evidence from the Demographic and Health Survey 2017–2018

**DOI:** 10.1186/s12884-023-05778-0

**Published:** 2023-06-22

**Authors:** Farjana Misu, Khurshid Alam

**Affiliations:** 1 Murdoch Business School, Murdoch University, Perth, WA 6150 Australia; 2 Department of Statistics, Jagannath University, Dhaka, 1100 Bangladesh

**Keywords:** Inequality, Equity strata, Postnatal care, Demographic and Health Survey, Bangladesh, Pakistan

## Abstract

**Background:**

Inequality in postnatal care (PNC) has remained a challenge in many low- and middle-income countries, like Bangladesh and Pakistan. The study examines within-country and between-country inequality in utilizing PNC services for Bangladesh and Pakistan.

**Methods:**

The study used the latest Demographic and Health Survey (DHS, 2017–2018) datasets of Bangladesh and Pakistan for women aged 15–49 years who had given at least one live birth in the three years preceding the survey. As outcome variables, three PNC service indicators were considered: PNC check of women, PNC check of newborns, and adequate PNC content of newborns. Concentration curves and equiplots were constructed to visually demonstrate inequality in PNC services. For ordered equity strata with more than two categories, the relative concentration index (RCI), absolute concentration index (ACI), and slope index of inequality (SII) were calculated to measure inequalities in the utilization of PNC services. For two categories equity strata, rate ratio (RR) and rate difference (RD) were calculated.

**Results:**

In Bangladesh, the level of inequality was high and almost the same for the PNC check of women and newborns based on women’s education (PNC women- RCI: 0.404, ACI: 0.403, SII: 0.624; and PNC newborn- RCI: 0.402, ACI: 0.402, SII: 0.622), wealth (PNC women- RCI: 0.448, ACI: 0.448, SII: 0.643; and PNC newborn- 0.441, ACI: 0.441, SII: 0.633), and number of ANC visits (PNC women- RCI: 0.329, ACI: 0.329, SII: 0.595; and PNC newborn- RCI: 0.329, ACI: 0.329, SII: 0.594). In Pakistan, the level of inequality was higher for the PNC check of women among all PNC services based on women’s education (ACI: 0.388 and SII: 0.676) and wealth (ACI: 0.397 and SII: 0.598). For Bangladesh and Pakistan, RR values (2.114 and 3.873, respectively) indicated greater media exposure-related inequality in adequate PNC content of newborns. Inequality in facility delivery was highest for PNC checks of women and newborns in Bangladesh (PNC women- RD: 0.905, PNC newborn- RD: 0.900) and Pakistan (PNC women- RD: 0.726, PNC newborn-RD: 0.743).

**Conclusion:**

Inequality was higher in Bangladesh than in Pakistan for PNC checks of women and newborns based on wealth, media exposure, and mode of delivery. For adequate PNC content of newborns, inequality was greater in Pakistan than in Bangladesh. Country-specific customized policies would better minimize the gap between the privileged and underprivileged groups and reduce inequality.

**Supplementary Information:**

The online version contains supplementary material available at 10.1186/s12884-023-05778-0.

## Background

Globally, the maternal mortality ratio (MMR) dropped by about 38% from 2000 to 2017 [1]. Despite this decline, low- and middle-income countries (LMICs) experienced around 94% of global maternal deaths, while nearly 20% accounted for South Asia [1]. Globally, the neonatal mortality rate (NMR) fell by 49% from 1990 to 2017 [2]; nevertheless, 99% occurred in LMICs [3]. More than half of neonatal deaths occur within the first two days of birth, and three-quarters within the first week [4]. In LMICs, the risk of women dying during the postpartum period is significantly higher [1]. Preterm delivery problems, birth asphyxia, and sepsis account for more than 75% of all neonatal deaths, while postpartum hemorrhage, hypertensive disorders, and infections account for the majority of maternal deaths [5]. Thus, the postpartum phase is critical, with implications for maternal and newborn health and survival.

Bangladesh and Pakistan are among the least three South Asian countries which experienced high rates of maternal deaths (173 per 100,000 and 140 per 100,000, respectively) in 2017, which are a long way from the SDG target of fewer than 70 deaths per 100,000 live births by 2030 [1, 6]. Bangladesh had an NMR of 30 per 1,000 live births [7], while Pakistan had an NMR of 42 per 1,000 live births in 2017 [8]. To achieve SDG target 3.2 (i.e., reducing neonatal deaths to at least 12 per 1,000 live births), both countries need to reduce neonatal deaths significantly [3]. Previous research reveals that if routine postnatal care (PNC) and curative care after deliveries are available to 90% of newborns and their mothers, 10–27% of newborn deaths can be avoided [9]. According to studies conducted in South Asian nations, a lack of appropriate, suitable, or prompt PNC increases morbidity and mortality [10, 11].

Empirical evidence shows that socio-economic and cultural barriers limit women’s access to PNC services. Low PNC usage is linked to poverty, education, and healthcare access [12]. According to a study in African countries, the utilization of PNC content in newborns was lowest among uneducated, impoverished mothers and those who had fewer than four ANC visits [13]. Women’s empowerment, mobile phone use throughout pregnancy, four or more ANC visits, skilled birth attendance, and pregnancy complications increased PNC checks among women in Bangladesh [14–16]. In Bangladesh, maternal age, rural residency, having no education and media exposure, multiparity, poor wealth status, husbands with no education, and husband’s employment status impede healthcare-seeking throughout pregnancy, delivery, and PNC [17, 18]. Lack of empowerment, awareness, mobility, transportation issues, and healthcare costs adversely affected PNC utilization in Pakistan [19, 20]. Higher birth order and birth interval, women with no formal education, and those residing in regional areas were significantly associated with the non-utilization of PNC services in Pakistan [21]. Thus, to improve PNC utilization and reduce maternal and child morbidity and mortality rates, countries need to focus more on reducing inequalities between population groups [22].

Bangladesh and Pakistan both suffer from high levels of poverty and have low economic growth rates [23]. Before 1971, Bangladesh and Pakistan shared a single entity known as East Pakistan and West Pakistan. Both nations shared common socio-political, religious, cultural, and economic backgrounds. Political, regional, and socio-economic disparities resulted in an independent Bangladesh from Pakistan in 1971 [24]. Now, after 50 years of separation, an investigation of PNC utilization and comparison within and between the two countries would be an interesting exercise and ‘food for thought’ for the health policymakers in the region. Additionally, among the South Asian countries, only Bangladesh and Pakistan have the same period Demographic and Health Survey (DHS, 2017–2018) datasets to compare.

Several Asian [6, 16] and African [25] studies have examined inequality in antenatal care (ANC), delivery care, and other maternal healthcare services. Few studies have measured wealth, education, and urban-rural inequality in PNC of women [12, 26, 27]. However, studies comprehensively measuring inequality in PNC of both women and newborns based on common equity strata are rare. Also, to our knowledge, no such study attempts to identify and compare within-country and between-country inequality in PNC services utilization between Bangladesh and Pakistan for the same period. The current study is an effort to help minimize this knowledge gap. Hence, our study examines and compares inequality in three PNC services using the latest and same period DHS, 2017–2018 datasets and applying relative and absolute inequality measures in common equity strata for Bangladesh and Pakistan. The findings of this study will help health policymakers and program managers to prioritize resource allocation in maternal and child health service interventions and meet SDG targets to reduce maternal and neonatal deaths.

## Methods

### Data

Study data were compiled from the latest DHS datasets of Bangladesh and Pakistan, collected during 2017–2018. These nationally representative cross-sectional surveys provided quality data on population, health, and nutrition to assist in evidence-based policymaking [7, 8]. These surveys used a stratified two-stage cluster sampling to draw a representative sample, and the survey report describes the survey design and data collection process [7, 8]. Ethical clearance to conduct the Bangladesh Demographic and Health Survey (BDHS) 2017–2018 was obtained from the National Research Ethics Committee of the Bangladesh Medical Research Council (Dhaka, Bangladesh) and ICF Macro Institutional Review Board (USA). The Pakistan Demographic and Health Survey (PDHS) 2017–2018 protocol was reviewed and approved by the National Bioethics Committee, Pakistan Health Research Council, and ICF Institutional Review Board.

### Sample size determination

**Fig. 1:**
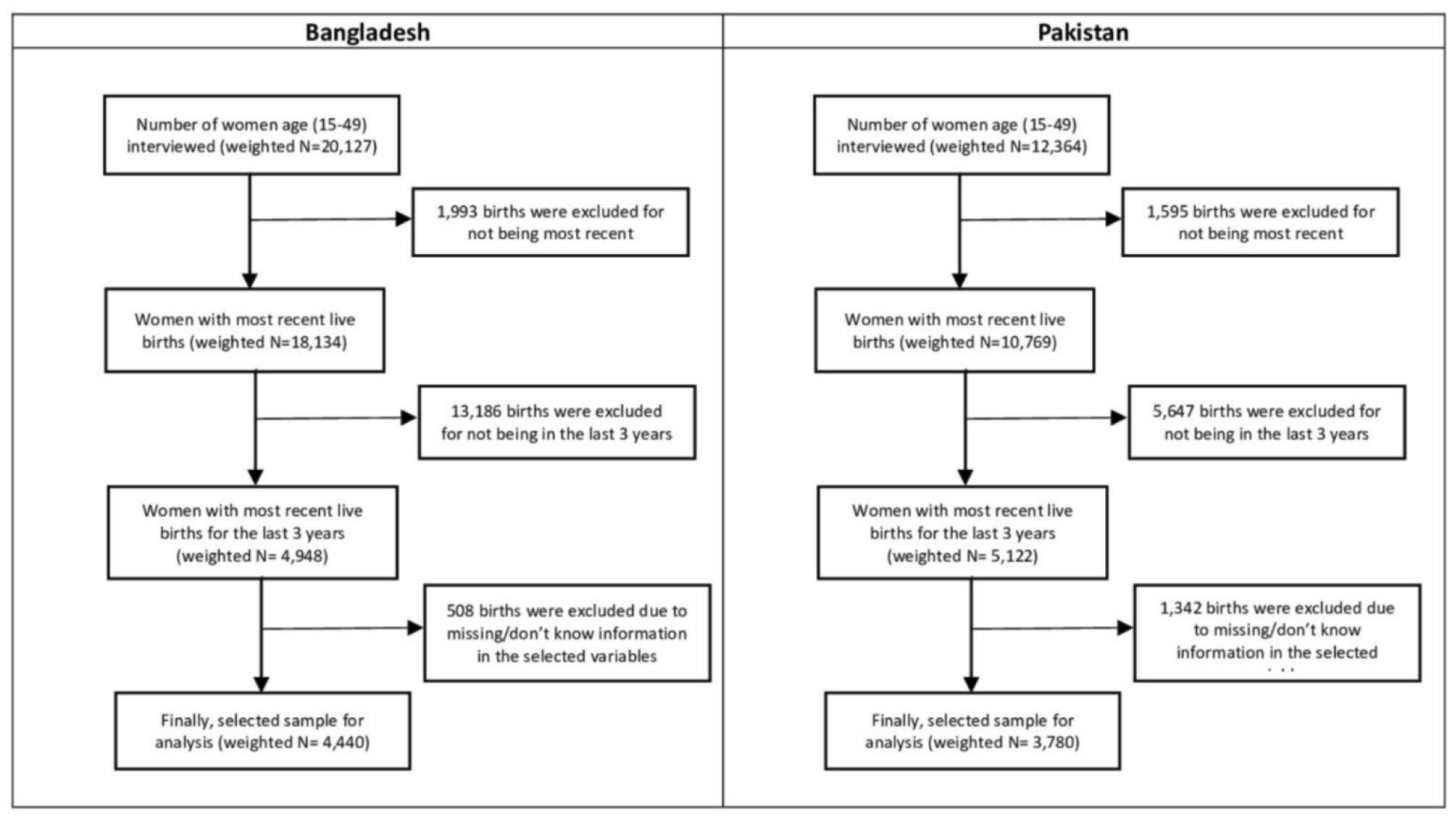
Sample Selection Procedure

BDHS successfully interviewed 20,127 women aged 15–49 years out of 20,376, resulting in a response rate of 98.8% [7]. The PDHS successfully interviewed 12,364 women aged 15–49 years out of a total of 13,118, yielding a response rate of 94.3% [8]. In the current study, we restricted our sample to women who had given birth to at least one live birth in the three years preceding the survey. When a woman had more than one live birth, we used the most recent live birth data. After excluding missing information from the selected outcome and independent variables, 4,440 women for Bangladesh and 3,780 women for Pakistan were retained for the final analysis. Figure 1 shows the sample selection procedure for Bangladesh and Pakistan.

### Outcome variables

The main outcome of interest of the study relates to the utilization of PNC for women and newborns. In this study, we assessed PNC services for women and newborns by three outcome variables: (i) PNC check of women within two days after birth by a skilled provider, (ii) PNC check of newborns within two days after birth by a skilled provider, and (iii) adequate PNC content of newborns within two days after birth.

### Equity strata

Based on existing literature [26, 28–32], we identified common equity strata (women’s age, women’s education, place of residence, women as household head, household size, employment status, wealth quintile, husband’s education, wanted last child, last live birth order, sex of last birth, pregnancy termination history, media exposure, women’s autonomy, facility delivery, mode of delivery, and number of ANC visits) to examine inequality in PNC service indicators. Subsequently, we conformed equity strata following statistical modelling. Supplementary Table 1 provides the categorization, labeling, and coding of the outcome variables and equity strata.

### Statistical analysis

We carried out the empirical analyses in four different steps. First, we undertook descriptive analyses of the background characteristics of women who had given birth to at least one live birth in the three years preceding the survey. We performed simple logistic regression models to evaluate the equity strata associated with our selected PNC service outcome indicators. Equity strata exhibiting a significant association with PNC services in simple logistic models were entered into multiple logistic regression models. Variance Inflation Factor (VIF) was used to evaluate the possible collinearity among independent variables before undertaking multiple logistic regression models. However, there was no multicollinearity problem among the independent variables. The level of statistical significance using *p*-values was set at *p*$\le$0.05. We calculated the utilization percentage of PNC indicators for each significant equity stratum. Finally, we employed relative and absolute inequality measures to identify inequality in PNC services based on equity strata, which are significantly associated with PNC utilization outcome indicators for Bangladesh or Pakistan.

To identify patterns of inequality, including linear, top, and bottom inequality, we employed equiplots, which display the distance in PNC coverage between different equity strata [33]. A description of equiplots is given in the Additional file 1. Since women’s age, women’s education, wealth quintile, and number of ANC visits are ordered equity strata with more than two categories; we employed the concentration curve, relative concentration index (RCI), absolute concentration index (ACI), and slope index of inequality (SII) to examine inequality in PNC services [34]. Absolute inequality draws attention to the actual disparity in coverage between two extreme groups and the actions needed to minimize the gap. The degree of injustice between the privileged and the underprivileged is shown by relative inequality [33]. Inequality measures are described in detail in the Additional file 1.

In the concentration curve, the line from the origin indicates perfect equality. The degree of inequality increases with the concentration curve’s distance from the line of perfect equality [35]. If the PNC service indicator is concentrated among the privileged, the concentration curve is below the line of perfect equality; if it is concentrated among the underprivileged, it is above the line of perfect equality. RCI, ACI, and SII take the value zero if there is no inequality. Greater absolute values indicate higher levels of inequality. Positive values indicate a concentration of the indicator among the privileged, and negative values indicate a concentration of the indicator among the underprivileged [36].

We used rate difference (RD) and rate ratio (RR) to measure absolute and relative inequality for ordered/non-ordered equity strata with two categories, such as place of residence, media exposure, women’s autonomy, mode of delivery, and facility delivery [34]. RR takes only positive values. The further the value of RR from 1, the higher the level of inequality. For RD, the larger the absolute value, the higher the level of inequality [36]. A description of RD and RR is given in the Additional file 1. All the statistical analyses were performed using STATA version 17.0. All analyses were weighted to consider complex survey design, using the *svy* command in Stata.

## Results

### Background characteristics

Table 1 presents the background characteristics of women aged 15–49 years who had given birth to at least one live birth in the three years preceding the survey for Bangladesh and Pakistan. In Bangladesh, around 76% of women were in the 20–34 years age category, and the majority lived in rural (65.70%) areas. The completion of secondary or higher education for women and their husbands was around 24% and 25%, respectively. More than half of the women had media exposure (54%) and decision-making autonomy (55%). Around half of the births (47%) were delivered in a health facility, and 37% of women had more than four ANC visits. In Pakistan (Table 1), most women (76%) were in the 20–34 years age category and lived in rural (57%) areas. The completion of secondary or higher education for women and their husbands was around 21% and 39%, respectively. Around 44% of women had media exposure, and 25% had decision-making autonomy. Among births, 60% were delivered in a health facility, and 32% of women had more than four ANC visits.

**Table 1:** Background Characteristics of Women with At least One Live Birth in the Last 3 Years

Background Characteristics	Bangladesh		Pakistan	
	Frequency (N = 4440)	*Percent*	Frequency (N = 3780)	*Percent*
**Women’s Age (years)**				
15–19	772	17.39	219	5.79
20–34	3390	76.35	2879	76.16
35–49	278	6.26	682	18.05
**Women’s Education**				
No formal education	278	6.26	2183	57.75
Primary education not completed	756	17.03	159	4.21
Primary education completed	465	10.47	299	7.91
Junior school completed	1864	41.98	329	8.70
Secondary or higher	1077	24.26	810	21.43
**Place of Residence**				
Urban	1523	34.30	1625	42.99
Rural	2917	65.70	2155	57.01
**Woman as Household Head**				
Yes	538	12.12	321	8.49
No	3902	87.88	3459	91.51
**Household Size**				
1–5 members	2235	50.34	729	19.29
6 or more members	2205	49.66	3051	80.71
**Employment Status**				
Currently employed	1678	37.79	425	11.24
Not currently employed	2762	62.21	3355	88.76
**Wealth Quintile**				
Poorest	970	21.85	998	26.40
Poorer	901	20.29	814	21.53
Middle	795	17.91	681	18.02
Richer	863	19.44	611	16.16
Richest	911	20.51	676	17.89
**Husband’s Education**				
No formal education	618	13.92	1213	32.09
Primary education not completed	837	18.85	176	4.66
Primary education completed	643	14.48	353	9.34
Junior school completed	1213	27.32	576	15.24
Secondary or higher	1129	25.43	1462	38.67
**Wanted Last Child**				
Yes	3493	78.67	3384	89.52
No	947	21.33	396	10.48
**Last Live Birth Order**				
First	1692	38.11	783	20.71
Second	1449	32.64	816	21.59
Third	748	16.84	628	16.62
Fourth or higher	551	12.41	1553	41.08
**Sex of Last Birth**				
Male	2327	52.41	1893	50.08
Female	2113	47.59	1887	49.92
**Pregnancy Termination History**				
Yes	742	16.71	1050	27.78
No	3698	83.29	2730	72.22
**Media Exposure**				
Yes	2398	54.01	1651	43.68
No	2042	45.99	2129	56.32
**Women’s Autonomy**				
Yes	2423	54.57	949	25.11
No	2017	45.43	2831	74.89
**Facility Delivery**				
Yes	2075	46.73	2250	59.52
No	2365	53.27	1530	40.48
**Mode of Delivery**				
Cesarean section	1392	31.35	567	15.00
Normal delivery	3048	68.65	3213	85.00
**Number of ANC Visits**				
Zero	372	8.38	715	18.91
1 to 4	2445	55.07	1843	48.76
More than four	1623	36.55	1222	32.33

The simple logistic regression models assessed the impact of each equity stratum on each outcome variable for Bangladesh (Supplementary Table 2) and Pakistan (Supplementary Table 3). Equity strata exhibiting a significant impact on PNC outcome variables in simple logistic models were entered into multiple logistic regression models (Supplementary Tables 2 & 3). Women’s age, education, place of residence, wealth quintile, media exposure, women’s autonomy, facility delivery, mode of delivery, and number of ANC visits had a significant (p < 0.05) impact on PNC outcome variables for Bangladesh or Pakistan after controlling the potential confounders.

### Coverage probability

Table 2 exhibits the coverage of PNC services for women and newborns by common equity strata for Bangladesh and Pakistan, where we observed considerable differences between and within the two countries. Both countries show an intra-country disparity in PNC by education attainments by women - as the education level of the women increases, the level of PNC utilization increases. For PNC check of women, the disparity was wider in Bangladesh (about 55% for the secondary or higher education of women than for no formal education) than in Pakistan (46% for the secondary or higher education of women than for no formal education). In Bangladesh, the disparity was also wider for PNC check of newborns (about 54% for the secondary or higher education of women than for no formal education) than in Pakistan (43% for the secondary or higher education of women than for no formal education).

The urban-rural disparity in PNC services was more prominent in Pakistan than in Bangladesh - the urban-rural gap in the PNC check of women and newborns differs by 26% and 25%, respectively, in Pakistan, whereas this gap was same and around 21% for both women and newborns in Bangladesh. Like education attainment, as wealth increases, the level of PNC utilization increases in Bangladesh and Pakistan. Nevertheless, within the country, the rich-poor disparity in PNC check of women and newborns was higher in Bangladesh (about 57% higher for the richest than the poorest for both women and newborns) than in Pakistan (50% for women and 47% for newborns). Additionally, the rich-poor disparity in adequate PNC content of newborns was higher in Pakistan (23%) than in Bangladesh (18%).

PNC utilization (Table 2) for women and newborns increased with media exposure in Bangladesh (around 27% increased for both women and newborns) and Pakistan (around 23% increased for both women and newborns). By facility delivery, the PNC check of women and newborns was higher in Bangladesh (around 91% higher for facility delivery than others for both women and newborns) than in Pakistan (73% higher for women and 74% higher for newborns). By mode of delivery, PNC utilization was higher in Bangladesh than in Pakistan. PNC utilization was consistently increasing with the number of ANC visits in Bangladesh and Pakistan, with mixed intra-country disparities between zero and more than four visits.

**Table 2:** Coverage of PNC Services by Common Equity Strata for Bangladesh and Pakistan

Equity Strata	Bangladesh			Pakistan		
	PNC Check of Women within 2 Days by Skilled Provider	PNC Check of Newborns within 2 Days by Skilled Provider	Adequate PNC Content of Newborns within 2 days	PNC Check of Women within 2 Days by Skilled Provider	PNC Check of Newborns within 2 Days by Skilled Provider	Adequate PNC Content of Newborns within 2 days
	*Percent* [95% CI]	*Percent* [95% CI]	*Percent* [95% CI]	*Percent* [95% CI]	*Percent* [95% CI]	*Percent* [95% CI]
**National**	49.36 [46.90-51.83]	49.42 [47.00-51.84]	13.13 [11.72–14.69]	48.37 [44.84–51.92]	51.25 [47.92–54.57]	8.00 [6.52–9.79]
**Women’s Age**						
15–19	51.42 [46.84–55.98]	51.77 [47.24–56.28]	10.91 [8.57–13.79]	40.63 [32.28–49.55]	47.29 [38.34–56.42]	1.84 [0.63–5.25]
20–34	49.24 [46.66–51.83]	49.28 [46.69–51.87]	13.95 [12.33–15.76]	50.05 [46.32–53.77]	52.97 [49.60-56.32]	8.21 [6.77–9.92]
35–49	44.68 [38.25–51.29]	44.03 [37.61–50.67]	9.33 [6.16–13.89]	43.26 [37.18–49.56]	44.76 [38.79–50.89]	8.98 [5.70-13.86]
**Women’s Education**						
No formal education	22.74 [17.59–28.88]	23.16 [17.92–29.40]	4.40 [2.38–8.01]	33.11 [29.25–37.22]	37.23 [33.40-41.22]	2.35 [1.54–3.56]
Primary education not completed	27.84 [23.77–32.32]	26.99 [23.02–31.36]	7.24 [5.24–9.91]	42.89 [33.71–52.59]	50.61 [41.48–59.70]	2.83 [1.07–7.25]
Primary education completed	30.47 [25.49–35.96]	33.16 [28.15–38.58]	8.44 [6.10-11.56]	51.60 [44.87–58.27]	50.59 [43.82–57.34]	7.43 [4.49–12.07]
Junior school completed	51.30 [48.39–54.20]	51.03 [48.14–53.92]	11.93 [10.21–13.91]	63.50 [55.27–71.01]	63.44 [55.28–70.89]	13.66 [9.22–19.78]
Secondary or higher	77.49 [74.45–80.25]	77.55 [74.57–80.26]	24.25 [21.18–27.60]	78.64 [73.92–82.70]	80.35 [75.79–84.23]	20.75 [16.84–25.30]
**Place of Residence**						
Urban	64.88 [61.03–68.54]	64.38 [60.60-67.98]	17.86 [15.34–20.69]	66.55 [61.88–70.91]	68.85 [64.48–72.91]	16.74 [13.43–20.66]
Rural	43.74 [40.79–46.74]	44.00 [41.10-46.94]	11.42 [9.76–13.32]	40.71 [36.47–45.08]	43.82 [39.81–47.93]	4.31 [2.98–6.21]
**Wealth Quintile**						
Poorest	23.83 [20.46–27.57]	23.53 [20.22–27.21]	7.06 [5.11–9.67]	32.52 [26.37–39.33]	34.74 [28.95–41.02]	1.09 [0.55–2.15]
Poorer	35.40 [31.71–39.26]	36.17 [32.42–40.09]	9.06 [7.18–11.37]	32.08 [27.10–37.50]	35.01 [29.67–40.75]	2.68 [1.41–5.04]
Middle	49.24 [44.78–53.71]	49.95 [45.50-54.41]	11.13 [8.90-13.84]	43.70 [38.30-49.26]	50.61 [45.78–55.43]	5.89 [3.83–8.94]
Richer	58.58 [54.57–62.49]	59.01 [54.95–62.94]	14.09 [11.33–17.39]	65.01 [58.56–70.95]	66.97 [60.61–72.77]	11.87 [7.64–17.99]
Richest	82.09 [79.13–84.72]	80.75 [77.63–83.53]	24.90 [21.73–28.37]	82.46 [78.06–86.13]	82.17 [76.07–86.98]	24.22 [19.87–29.17]
**Media Exposure**						
Yes	61.23 [58.60-63.79]	61.53 [58.94–64.05]	17.21 [15.37–19.22]	60.89 [56.81–64.83]	63.52 [59.56–67.30]	13.25 [10.97–15.91]
No	34.82 [31.63–38.15]	34.58 [31.48–37.81]	8.14 [6.52–10.12]	37.45 [33.21–41.88]	40.55 [36.38–44.86]	3.42 [2.30–5.05]
**Women’s Autonomy**						
Yes	49.47 [46.48–52.47]	49.20 [46.25–52.16]	14.02 [12.22–16.03]	59.95 [54.99–64.71]	64.15 [59.65–68.41]	11.75 [9.04–15.14]
No	49.24 [46.14–52.34]	49.68 [46.63–52.73]	12.09 [10.33–14.10]	43.48 [39.78–47.28]	45.80 [42.17–49.47]	6.41 [5.00-8.19]
**Facility Delivery**						
Yes	97.96 [97.12–98.56]	97.73 [96.93–98.33]	25.71 [23.13–28.46]	76.31 [73.38–79.02]	79.85 [76.92–82.49]	12.72 [10.49–15.35]
No	7.41 [6.15–8.91]	7.71 [6.46–9.17]	2.28 [1.61–3.21]	3.74 [2.33–5.96]	5.57 [3.94–7.82]	0.45 [0.18–1.15]
**Mode of Delivery**						
Cesarean section	98.88 [98.01–99.37]	98.44 [97.56-99.00]	28.79 [25.69–32.10]	94.97 [92.26–96.76]	91.10 [87.48–93.74]	22.47 [18.49–27.03]
Normal delivery	26.87 [24.68–29.18]	27.15 [24.98–29.43]	6.02 [4.95–7.31]	37.36 [34.29–41.36]	42.18 [38.78–45.65]	4.70 [3.50–6.29]
**Number of ANC Visits**						
Zero	12.86 [8.90-18.22]	13.47 [9.47–18.82]	1.53 [0.49–4.65]	10.29 [7.46–14.02]	13.05 [9.53–17.60]	0.31 [0.07–1.35]
1 to 4	42.54 [39.79–45.34]	42.48 [39.74–45.27]	10.59 [9.12–12.25]	40.66 [36.74–44.71]	44.87 [41.08–48.72]	4.80 [3.42–6.69]
More than four	68.58 [65.54–71.47]	68.68 [65.61–71.61]	19.84 [17.45–22.47]	77.97 [74.48–81.12]	78.76 [74.96–82.12]	16.63 [13.43–20.43]

CI: Confidence interval

### Equiplots

**Fig. 2:**
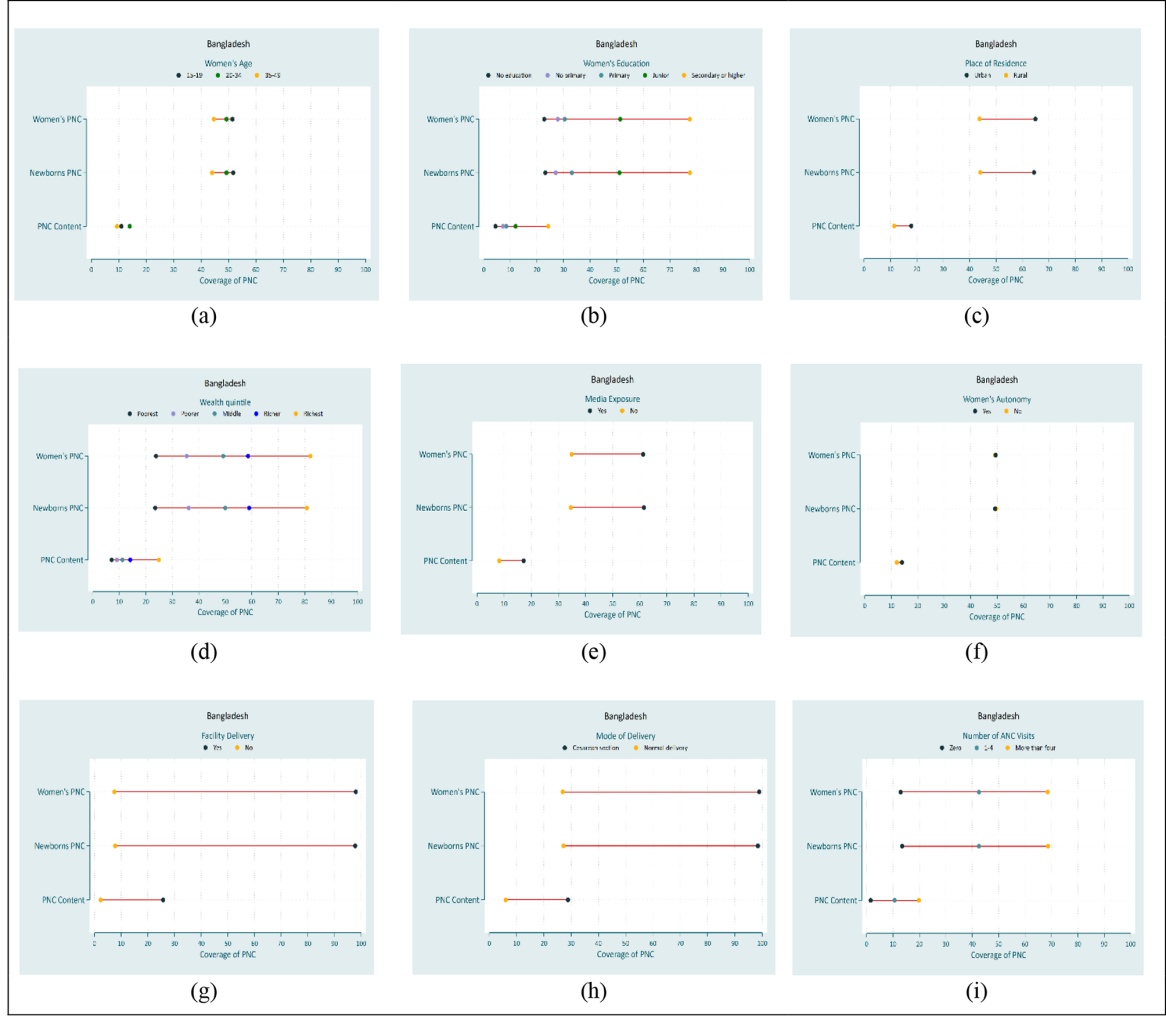
Equiplot of equity strata for PNC services in Bangladesh

**Fig. 3:**
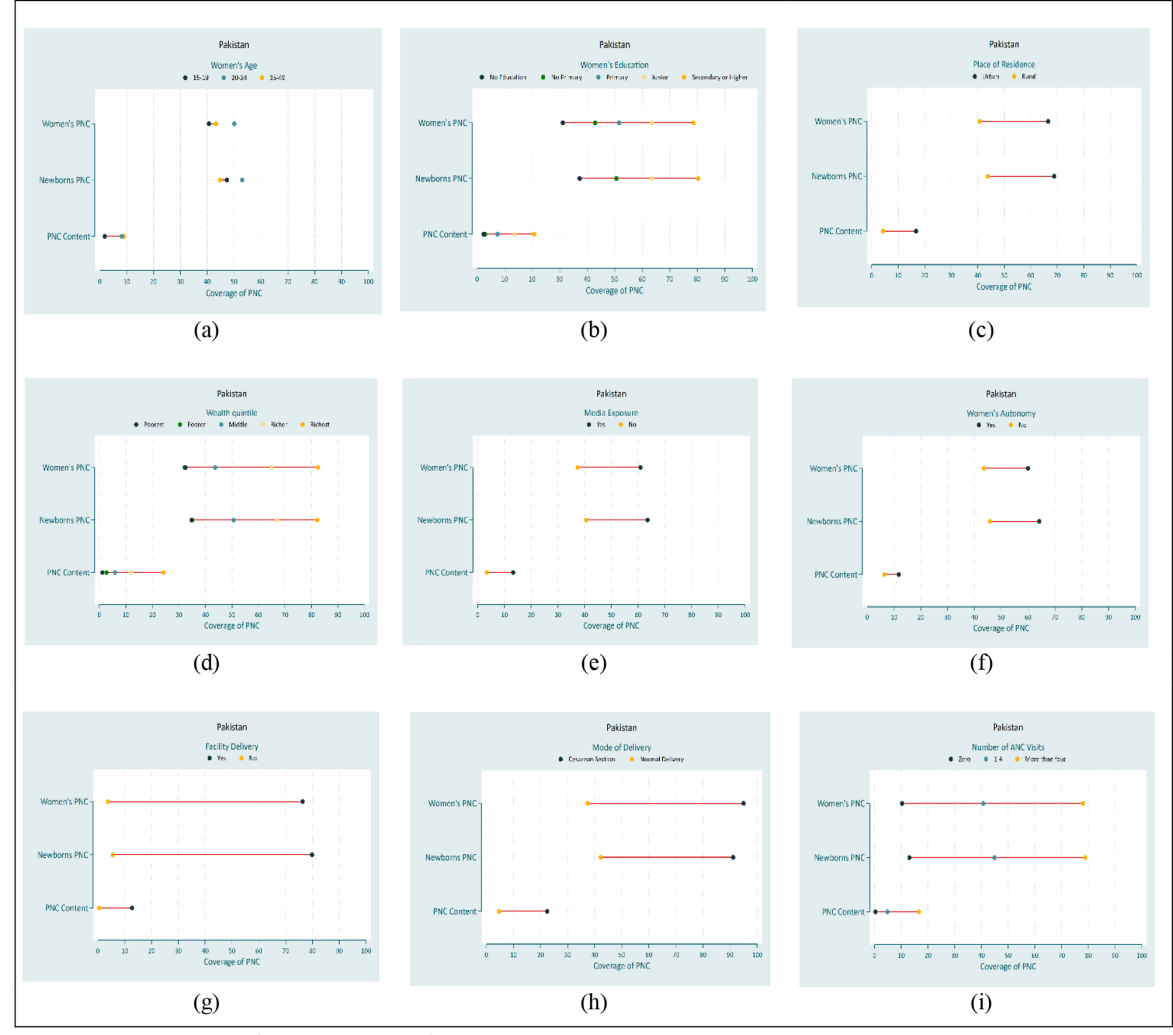
Equiplot of equity strata for PNC services in Pakistan

Figures 2 and 3 show equiplots of PNC services by common equity strata in Bangladesh and Pakistan. Inequality was present among most of the equity strata in Bangladesh and Pakistan. In Bangladesh, there was top inequality indicating the widest gap exists for the secondary or higher educated women (Fig. 2b) and the richest wealth quintile (Fig. 2d) in all PNC service indicators. In Pakistan, there was top inequality indicating the widest gap exists for the richest wealth quintile (Fig. 3d) and more than four ANC visits (Fig. 3i) in all PNC service indicators.

### Concentration curves

**Fig. 4:**
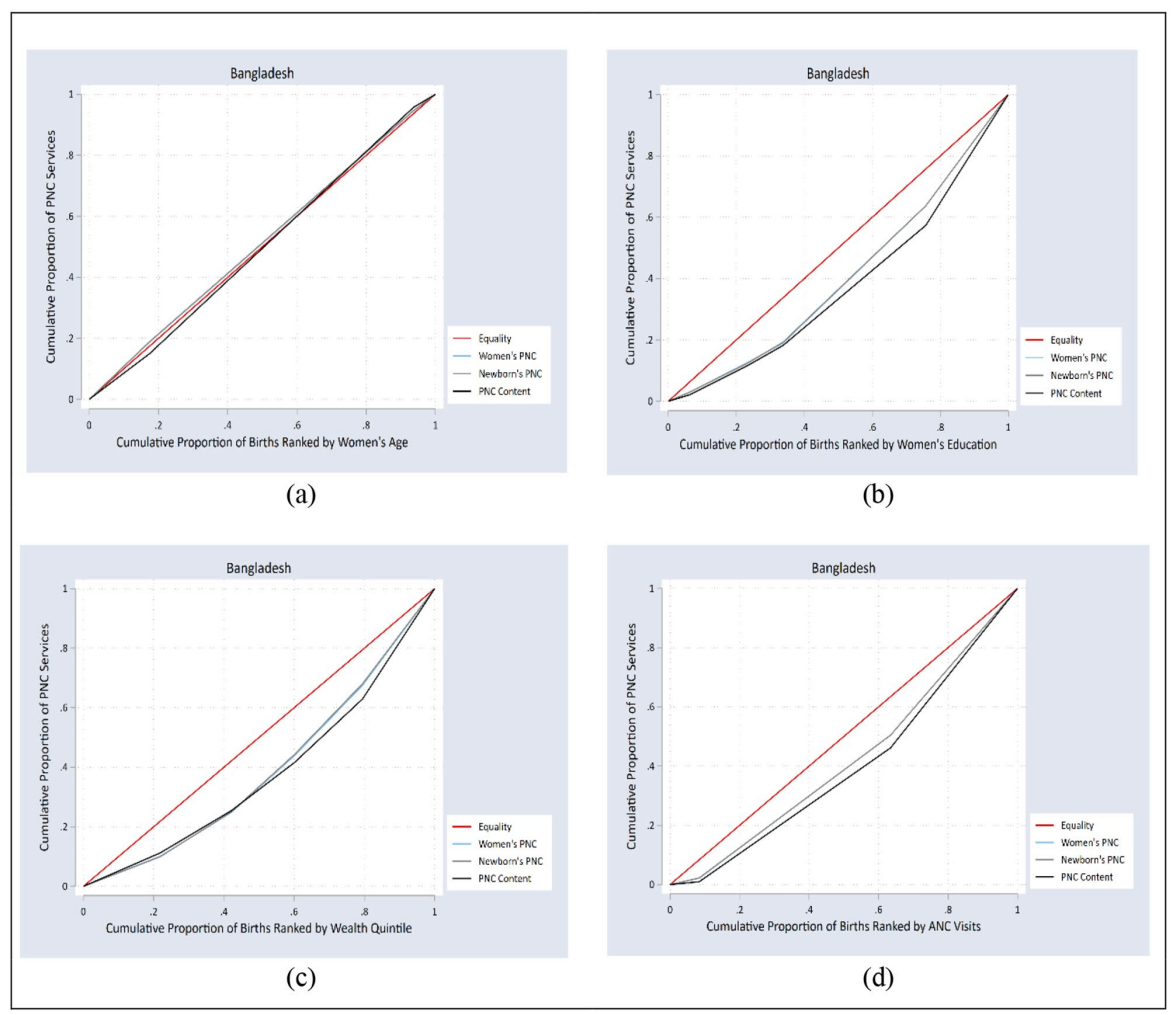
Concentration curve of equity strata for PNC services in Bangladesh

**Fig. 5:**
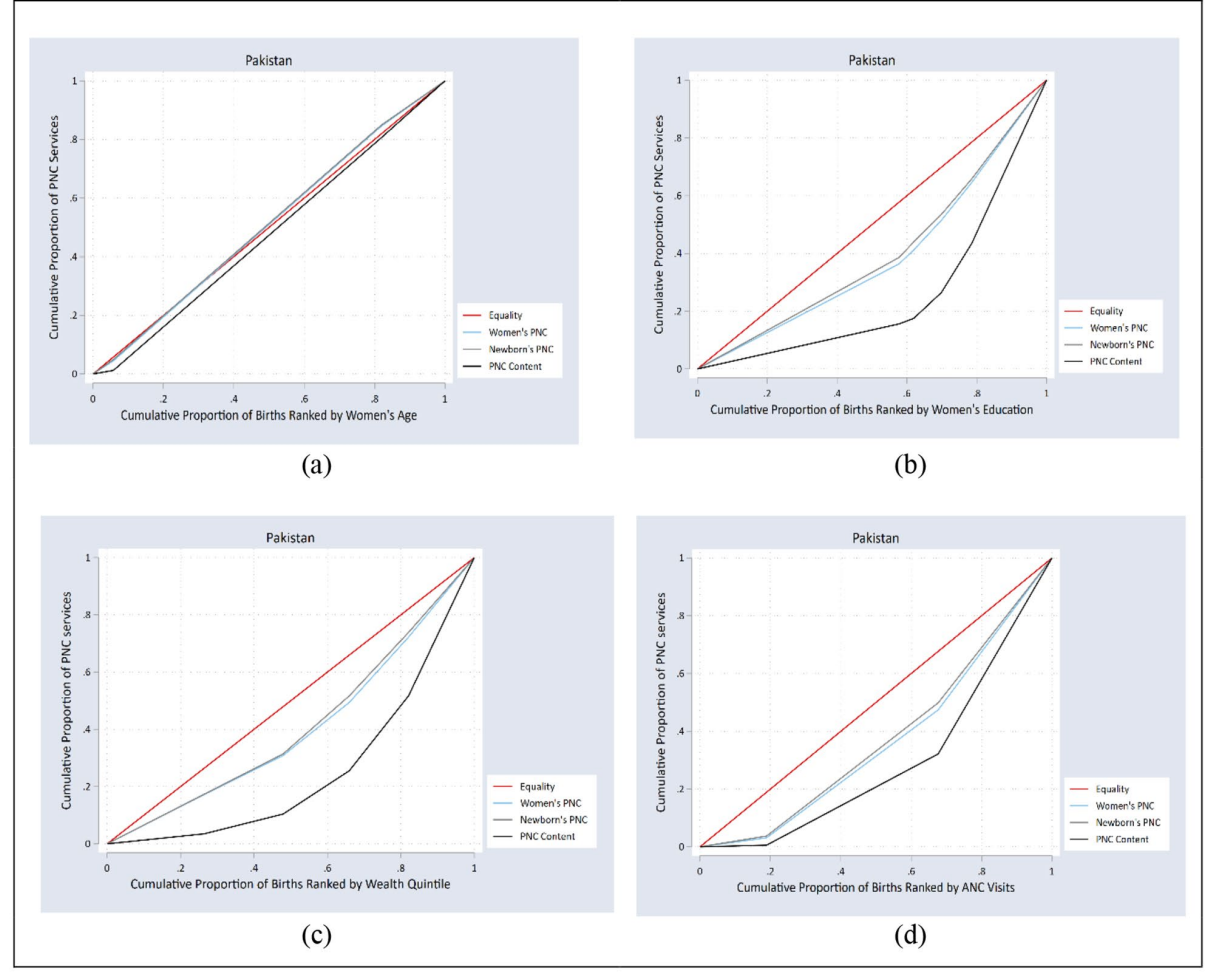
Concentration curve of equity strata for PNC services in Pakistan

Figures 4 and 5 represent concentration curves for all PNC services by women’s age, education, wealth, and number of ANC visits for Bangladesh and Pakistan. The concentration curve close to the line of equality in Figs. 4a and 5a show no inequality in the utilization of PNC based on women’s age in Bangladesh and Pakistan, respectively. The concentration curves of all the PNC indicators under the line of equality imply that inequality was disproportionately concentrated in the women who completed secondary or higher education (Figs. 4b and 5b), women in the richest wealth quintile (Figs. 4c and 5c), and women who had more than four ANC visits (Figs. 4d and 5d) for both Bangladesh and Pakistan.

### Inequality measures

Table 3 shows the relative and absolute measure of inequality for PNC services based on women’s age, education, wealth, and number of ANC visits for Bangladesh and Pakistan. For both countries, women’s education, wealth, and number of ANC visits had significant inequality ($p\le 0.05$) for all inequality measures (RCI, ACI, and SII) in all the PNC services. In Bangladesh, the values of RCI, ACI, and SII suggest that the level of inequality was high and almost same for the PNC check of women and newborns based on women’s education (PNC women- RCI: 0.404 [95% CI: 0.370–0.437], ACI: 0.403 [95% CI: 0.370–0.437], SII: 0.624 [95% CI: 0.579–0.670]; and PNC newborn- RCI: 0.402 [95% CI: 0.369–0.435], ACI: 0.402 [95% CI: 0.369–0.435], SII: 0.622 [95% CI: 0.577–0.666]), wealth (PNC women- RCI: 0.448 [95% CI: 0.413–0.483], ACI: 0.448 [95% CI: 0.413–0.483], SII: 0.643 [95% CI: 0.600-0.685]; and PNC newborn- 0.441 [95% CI: 0.406–0.475], ACI: 0.441 [95% CI: 0.406–0.475], SII: 0.633 [95% CI: 0.591–0.676]), and number of ANC visits (PNC women- RCI: 0.329 [95% CI: 0.295–0.364], ACI: 0.329 [95% CI: 0.295–0.363], SII: 0.595 [95% CI: 0.543–0.648]; and PNC newborn- RCI: 0.329 [95% CI: 0.294–0.364], ACI: 0.329 [95% CI: 0.294–0.364], SII: 0.594 [95% CI: 0.540–0.648]). For women’s education, wealth status, and number of ANC visits, the values of ACI (0.134 [95% CI: 0.108–0.160], 0.130 [95% CI: 0.100-0.159], and 0.112 [95% CI: 0.090–0.135], respectively), and SII (0.227 [95% CI: 0.182–0.272], 0.201 [95% CI: 0.154–0.248], and 0.214 [95% CI: 0.172–0.256], respectively) revealed a lower level of inequality in adequate PNC content of newborns in Bangladesh. In Pakistan, according to the values of ACI and SII (Table 3), the level of inequality was higher for the PNC check of women among all PNC services based on women’s education (ACI: 0.388 [95% CI: 0.333–0.444] and SII: 0.676 [95% CI: 0.605–0.747]), and wealth (ACI: 0.397 [95% CI: 0.331–0.462] and SII: 0.598 [95% CI: 0.514–0.681]). For women’s education, wealth status, and number of ANC visits, the values of ACI (0.152 [95% CI: 0.117–0.187], 0.164 [95% CI: 0.131–0.198], and 0.127 [95% CI: 0.095–0.159], respectively) and SII (0.270 [95% CI: 0.208–0.333], 0.286 [95% CI: 0.224–0.347], and 0.236 [95% CI: 0.174–0.298], respectively) revealed a lower level of inequality in adequate PNC content of newborns in Pakistan.

**Table 3:** Relative and Absolute Inequality Index of PNC services by Common Equity Strata

Inequality measures	Bangladesh			Pakistan		
	PNC Check of Women within 2 Days by Skilled Provider	PNC Check of Newborns within 2 Days by Skilled Provider	Adequate PNC Content of Newborns within 2 days	PNC Check of Women within 2 Days by Skilled Provider	PNC Check of Newborns within 2 Days by Skilled Provider	Adequate PNC Content of Newborns within 2 days
**Women’s Age**						
RCI [95% CI]	-0.023 [(-0.052)-0.006], *p*-value = 0.12	-0.026 [(-0.055)-0.003], *p*-value = 0.08	0.017 [(-0.021)-0.054], *p*-value = 0.38	-0.020 [(-0.055)-0.016], *p*-value = 0.28	-0.035 [(-0.069) -(-0.002)], *p*-value = 0.04	0.059 [(-0.014)-0.131], *p*-value = 0.11
ACI [95% CI]	-0.023 [(-0.052)-0.006], *p*-value = 0.12	-0.026 [(-0.055)-0.003], *p*-value = 0.08	0.008 [(-0.009)-0.025], *p*-value = 0.38	-0.020 [(-0.055)-0.016], *p*-value = 0.28	-0.035 [(-0.069) -(-0.002)], *p*-value = 0.04	0.017 [(-0.004)-0.039], *p*-value = 0.11
SII [95% CI]	-0.059 [(-0.135)-0.017], *p*-value = 0.18	-0.068 [(-0.145)-0.009], *p*-value = 0.08	0.024 [(-0.023)-0.070], *p*-value = 0.32	-0.038 [(-0.142)-0.066], *p*-value = 0.48	-0.087 [(-0.185)-0.010], *p*-value = 0.08	0.056 [0.001–0.112], *p*-value = 0.05
**Women’s Education**						
RCI [95% CI]	0.404 [0.370–0.437], *p*-value = 0.00	0.402 [0.369–0.435], *p*-value = 0.00	0.294 [0.237–0.351], *p*-value = 0.00	0.389 [0.333–0.444], *p*-value = 0.00	0.360 [0.305–0.415], *p*-value = 0.00	0.516 [0.396–0.635], *p*-value = 0.00
ACI [95% CI]	0.403 [0.370–0.437], *p*-value = 0.00	0.402 [0.369–0.435], *p*-value = 0.00	0.134 [0.108–0.160], *p*-value = 0.00	0.388 [0.333–0.444], *p*-value = 0.00	0.360 [0.305–0.414], *p*-value = 0.00	0.152 [0.117–0.187], *p*-value = 0.00
SII [95% CI]	0.624 [0.579–0.670], *p*-value = 0.00	0.622 [0.577–0.666], *p*-value = 0.00	0.227 [0.182–0.272], *p*-value = 0.00	0.676 [0.605–0.747], *p*-value = 0.00	0.647 [0.571–0.722], *p*-value = 0.00	0.270 [0.208–0.333], *p*-value = 0.00
**Wealth Status**						
RCI [95% CI]	0.448 [0.413–0.483], *p*-value = 0.00	0.441 [0.406–0.475], *p*-value = 0.00	0.284 [0.219–0.349], *p*-value = 0.00	0.397 [0.332–0.462], *p*-value = 0.00	0.386 [0.321–0.450], *p*-value = 0.00	0.558 [0.444–0.673], *p*-value = 0.00
ACI [95% CI]	0.448 [0.413–0.483], *p*-value = 0.00	0.441 [0.406–0.475], *p*-value = 0.00	0.130 [0.100-0.159], *p*-value = 0.00	0.397 [0.331–0.462], *p*-value = 0.00	0.385 [0.321-450], *p*-value = 0.00	0.164 [0.131–0.198], *p*-value = 0.00
SII [95% CI]	0.643 [0.600-0.685], *p*-value = 0.00	0.633 [0.591–0.676], *p*-value = 0.00	0.201 [0.154–0.248], *p*-value = 0.00	0.598 [0.514–0.681], *p*-value = 0.00	0.585 [0.450–0.670], *p*-value = 0.00	0.286 [0.224–0.347], *p*-value = 0.00
**Number of ANC Visits**						
RCI [95% CI]	0.329 [0.295–0.364], *p*-value = 0.00	0.329 [0.294–0.364], *p*-value = 0.00	0.246 [0.197–0.296], *p*-value = 0.00	0.481 [0.444–0.518], *p*-value = 0.00	0.458 [0.417–0.498], *p*-value = 0.00	0.430 [0.321–0.539], *p*-value = 0.00
ACI [95% CI]	0.329 [0.295–0.363], *p*-value = 0.00	0.329 [0.294–0.364], *p*-value = 0.00	0.112 [0.090–0.135], *p*-value = 0.00	0.480 [0.444–0.517], *p*-value = 0.00	0.457 [0.417–0.498], *p*-value = 0.00	0.127 [0.095–0.159], *p*-value = 0.00
SII [95% CI]	0.595 [0.543–0.648], *p*-value = 0.00	0.594 [0.540–0.648], *p*-value = 0.00	0.214 [0.172–0.256], *p*-value = 0.00	0.777 [0.734–0.821], *p*-value = 0.00	0.755 [0.704–0.806], *p*-value = 0.00	0.236 [0.174–0.298], *p*-value = 0.00

RCI: Relative Concentration Index; ACI: Absolute Concentration Index; SII: Slope Index of inequality; CI: Confidence interval

Table 4 shows relative (RR) and absolute (RD) inequality measures of PNC services by common equity strata for Bangladesh and Pakistan. For Bangladesh, the RR (1.563) indicated a more significant urban-rural inequality in adequate PNC content of newborns, while RD (0.211) indicated greater urban-rural inequality in the PNC check of women among all PNC service indicators. For Pakistan, the level of urban-rural inequality was greater for adequate PNC content of newborns according to the RR (3.879), while according to RD (0.233), it was greater and almost the same for PNC checks of women (RD: 0.258) and newborns (RD: 0.250). For Bangladesh and Pakistan, RR values (2.114 and 3.873, respectively) indicated greater media exposure-related inequality in adequate PNC content of newborns. According to RR values (1.833), women’s autonomy-related inequality was greater in adequate PNC content of newborns for Pakistan. For Bangladesh and Pakistan, the mode of delivery-related inequality was highest in adequate PNC content of newborns (RR: 3.680, RR: 4.778, respectively), while RD values (0.720 and 0.572, respectively) indicated the highest inequality in PNC check for women. According to the RR values (13.212) for Bangladesh, facility delivery-related inequality was more significant in women’s PNC check. However, according to the values of RD, inequality in facility delivery was highest and almost the same for PNC checks of women and newborns in Bangladesh (PNC women- RD: 0.905, PNC newborn- RD: 0.900) and Pakistan (PNC women- RD: 0.726, PNC newborn-RD: 0.743).

**Table 4:** Relative and Absolute Inequality Measure of PNC Services by Common Equity Strata

Inequality measures	Bangladesh			Pakistan		
	PNC Check of Women within 2 Days by Skilled Provider	PNC Check of Newborns within 2 Days by Skilled Provider	Adequate PNC Content of Newborns within 2 days	PNC Check of Women within 2 Days by Skilled Provider	PNC Check of Newborns within 2 Days by Skilled Provider	Adequate PNC Content of Newborns within 2 days
**Urban-Rural**						
RR (ref: Urban)	1.483	1.463	1.563	1.635	1.571	3.879
RD (ref: Urban)	0.211	0.204	0.064	0.258	0.250	0.124
**Media Exposure**						
RR (ref: Yes)	1.759	1.779	2.114	1.626	1.566	3.873
RD (ref: Yes)	0.264	0.270	0.091	0.234	0.230	0.098
**Women’s Autonomy**						
RR (ref: Yes)	1.005	0.990	1.159	1.379	1.401	1.833
RD (ref: Yes)	0.002	-0.005	0.019	0.165	0.184	0.053
**Facility Delivery**						
RR (ref: Yes)	13.212	12.681	11.273	20.396	14.334	28.001
RD (ref: Yes)	0.905	0.900	0.234	0.726	0.743	0.123
**Mode of Delivery**						
RR (ref: Cesarean)	3.680	3.626	4.780	2.515	2.160	4.778
RD (ref: Cesarean)	0.720	0.713	0.228	0.572	0.489	0.178

RR: Rate ratio; RD: Rate difference; ref: reference category

## Discussion

Our study demonstrates intra-country and inter-country inequality in the utilization of PNC services (PNC check of women, PNC check of newborns, and adequate PNC content of newborns) by common equity strata using the latest DHS data of Bangladesh and Pakistan of the same year (2017–2018). The utilization of PNC checks of women and newborns were almost same in Bangladesh and Pakistan. For adequate PNC content of newborns, the utilization was lower in Bangladesh and Pakistan. Inequalities in PNC utilization by rich-poor, urban-rural, educational attainments, media exposure, women’s autonomy, mode of delivery, facility delivery, and number of ANC visits are prevalent in both Bangladesh and Pakistan. Inequality was higher in Bangladesh compared to Pakistan for PNC checks of women and newborns based on wealth, media exposure, and mode of delivery. For adequate PNC content of newborns, inequality was greater in Pakistan than in Bangladesh based on women’s education, wealth, residency, media exposure, autonomy, and facility delivery.

In Bangladesh, inequality in PNC check of women and newborns exists to a larger extent by rich-poor, women’s education, and number of ANC visits. In Pakistan, across different PNC service indicators, inequality was highest in PNC check of women by wealth, women’s education, and number of ANC visits. Studies from Rwanda [37], Pakistan [38], and Uganda [39] exhibit similar findings that wealthier women utilized PNC services more than poorer women. The economic status of women is very crucial in healthcare. Women of the richest wealth quintile have affordability for health services, while the poorest women can hardly afford out-of-pocket payments for any health emergencies [40, 41]. Thus, the low economic condition prevents women from accessing essential maternal healthcare services and instigates rich-poor inequality in society.

Several South Asian [38, 40] and African [42, 43] studies indicate that women with secondary or higher education utilize PNC services more than those with primary or below education. Educated women exhibit higher health awareness, health-seeking behavior, and better decision-making ability toward healthcare use than less educated groups [44, 45]. Consequently, the differences in perception about the importance and necessity of healthcare among higher educated and non-educated women increase the inequality in PNC services.

In Bangladesh and Pakistan, inequality was higher in the utilization of PNC services for number of ANC visits favoring more than four ANC visits. These findings are consistent with other studies conducted in Zimbabwe [46] and Malawi [47], which suggested that the utilization of PNC services is higher among women who had attended at least four ANC visits [46, 47]. This can be attributable to the fact that during the ANC visits, women were able to interact more with health workers who encouraged and counseled them to use PNC services to minimize health problems during pregnancy, delivery, and after childbirth [48].

There was urban-rural inequality in PNC services utilization in Bangladesh and Pakistan. These results also accord with earlier studies in Morocco [49] and Malawi [47], which found that urban women utilized PNC services more than rural women. Lack of transportation, long distances to health centers, and bad roads might restrict rural women from accessing PNC services [27, 41]. Thus, the difference in infrastructure and health facilities prompts urban-rural inequality.

Inequality was greater in PNC service indicators, favouring women who were exposed to media in Bangladesh and Pakistan. A study in South Asia [50] supports the same finding, where women exposed to media were more likely to utilize maternal healthcare services. Media has the potential to improve women’s knowledge of maternal health issues and encourage them to utilize maternal healthcare services [51].

In Bangladesh and Pakistan, inequality was present for women’s autonomy favouring women who had the autonomy to make decisions alone or jointly with their husbands. Previous research has also identified that women’s decision-making autonomy influences the utilization of maternal healthcare services [52]. Women with decision-making autonomy have more influence within their households and are more likely to use healthcare services for themselves and their children [53].

Inequality was greater in PNC services, favouring women who had facility delivery in Bangladesh and Pakistan. A review study in Ethiopia [54] and another study in rural Zambia [55] revealed that women who gave delivery in a health facility were more likely to use PNC services than those who gave birth at home. A plausible explanation for our finding may be that women who delivered in a healthcare facility were more exposed to maternal health education relevant to PNC services at the time of delivery and thus got opportunities to learn about the benefits and availability of PNC services [21, 56].

Inequality was greater for the mode of delivery favoring cesarean section delivery in receiving PNC services both in Bangladesh and Pakistan. These findings align with studies undertaken in Northwestern Ethiopia [57] and Rural Tanzania [58], where women were significantly more likely to use a health facility for PNC following cesarean delivery. This is due to the fact that mothers who deliver through cesarean section tend to be more susceptible to postpartum complications, and obtaining PNC as part of follow-up treatment would be the strategy to minimize these perceived complications [59, 60].

Similar to Bangladesh and Pakistan, the utilization of PNC content of newborns was minimal in countries of similar settings [61]. The probable reason for the low utilization of PNC content in Bangladesh could be the unavailability and inaccessibility of critical interventions during the PNC period for all population groups, as well as the lack of awareness among healthcare professionals regarding the significance of adequate PNC content of newborns [62]. As in other resource-constrained countries such as Rwanda [63], rural China [64], and Bangladesh [62], the lack of sufficient community awareness regarding PNC components and the lower emphasis given to some PNC components by healthcare/PNC providers could be a possible reason for low utilization of adequate PNC content of newborns in Pakistan as well.

Due to a considerable gap between privileged and underprivileged groups in utilizing PNC check of women and newborns, the inequality was higher in Bangladesh than in Pakistan by wealth status, media exposure, and mode of delivery. In Bangladesh, policies like cash transfers, voucher schemes, and removing user fees may not be adequately monitored to support vulnerable groups, resulting in increased inequality [65]. Again, inequality was greater in Pakistan than in Bangladesh for adequate PNC content of newborns based on women’s education, wealth, residency, media exposure, autonomy, and facility delivery. Lack of monitoring of health policies and health intervention programs like Public Private Partnerships (PPP), Basic Health Units, and Rural Health Centres may instigate inequality in PNC services in Pakistan [66].

The findings of this study suggest that the primary goal for policymakers should be to eradicate inequitable healthcare utilization across population groups [65]. The government program may need large-scale implementation of pro-poor policies and interventions to increase PNC utilization among low-income women, particularly in rural areas. Emphasis should also be made on designing public health interventions focusing on women’s education and household autonomy. Access to media should be enhanced to spread different health messages. ’However, combining social development programs with equity-oriented health policies could be a better solution to combat the inequality crisis (for example, Maldives incorporated a Master Health plan with social safety net) [67]. Evidence confirms that customized program policies or strategies work better when they come together with appropriate resource allocation [31]. The countries should increase the health budget for the vulnerable areas which experience inequitable access to quality PNC services. It is also instructive to comprehend the diversity of techniques that match a country’s political, economic, and cultural contexts.

Our study’s strength is that we compared inequality between two historically connected countries, Bangladesh and Pakistan, using extensive nationally representative surveys of the same period. In addition, we used relative and absolute measures to assess inequality in PNC services. Regardless of the study’s strengths, there are several limitations to note. Due to the use of a cross-sectional study design, causation assumptions could not be drawn in this investigation. As a result, the findings should be explained with caution. We admit that the data on PNC utilization were self-reported, which may not be free from bias. There is also a risk of recall bias due to including women who had a live birth three years preceding the survey. This bias could result in overestimating or underestimating the utilization of maternal healthcare services. To reduce this effect, the analysis was conducted on the most recent birth during the three years preceding the survey. However, more research based on other household survey data should be carried out to validate the findings of the current study.

## Conclusion

Inequality pertains to PNC services by common equity strata in Bangladesh and Pakistan. Although the utilization of PNC check of women and newborns were almost the same in Bangladesh and Pakistan, it was lower for adequate PNC content of newborns in both countries. The level of inequality was higher in Bangladesh compared to Pakistan for PNC checks of women and newborns based on wealth, media exposure, and mode of delivery. Furthermore, for adequate PNC content of newborns, inequality was greater in Pakistan than in Bangladesh. Therefore, focusing on improving the utilization of PNC services, including health equality indicators in national monitoring frameworks, and combining policies based on country context would be more effective in minimizing the gaps between the privileged and underprivileged groups.

## Electronic supplementary material

Below is the link to the electronic supplementary material.

Supplementary Material 1

Supplementary Material 2

## Data Availability

The dataset analyzed during the current study are available in the DHS Program website, https://dhsprogram.com/data/available-datasets.cfm.
