# Supplementary material for: Comparison of inequality in utilization of postnatal care services between Bangladesh and Pakistan: Evidence from the Demographic and Health Survey 2017–2018
Source: BMC Pregnancy Childbirth. 2023 Jun 22;23:461. doi: 10.1186/s12884-023-05778-0 (PMC10286509; doi:10.1186/s12884-023-05778-0)
Supplement: Supplementary file 1 — Supplementary Material 1 [file 12884_2023_5778_MOESM1_ESM.docx]

**Description of Inequality Measures:**

**Measuring inequality for ordered equity strata with more than two categories**

The concentration curve is a graphical representation of inequality in PNC use that allows comparison of the degree of inequality across time or between nations. The concentration curve compares the cumulative proportion of PNC use indicators to the cumulative proportion of people ranked by various equity strata (such as wealth quintile and education).

Although the concentration curve is a useful tool for graphing inequality, RCI, ACI and SII were employed to measure the magnitude of inequality in PNC use for common equity strata. Due to the concentration index's compliance with three criteria for a reliable socioeconomic inequality index, it is a widely used indicator of socioeconomic health inequalities. The index should be responsive to the sub-population group sizes, reflect health disparities resulting from socioeconomic features, and represent the entire population.

The RCI is based on the relative concentration curve, which is twice the area between the relative concentration curve and the perfect equality line. If the concentration curve is above (below) the line of equality, the RCI is negative (positive), indicating that the use of PNC services is concentrated among underprivileged (privileged) groups. The RCI index is calculated as follows:

where $h_{i}$ is the maternal healthcare variable of interest for $i^{th}$women, μ is the mean of the maternal healthcare use variable for the whole sample, $r_{i}= \frac{i}{N}$, is the fractional rank of $i^{th}$ women in the distribution from the underprivileged woman ($i$ = 1) to the privileged woman ($i$ = N), and $\sigma_{r}^{2}$ is the variance of fractional rank. The ordinary least squares (OLS) estimate of $\alpha_{1}$ is used to determine the RCI. Since our outcome variable of interest is binary, the minimum and maximum values of the RCI are not – 1 and + 1, thus, the RCI was normalized by multiplying the estimated index by $\frac{1}{1-\mu}$ . Absolute socioeconomic inequality in healthcare consumption is calculated using the generalized concentration index. Since the generalized concentration index does not satisfy this condition, the *Erreygers* modified the generalized/ ACI (hereafter the $ACI=RCI\times4\mu$) was used to calculate absolute inequality in healthcare use.

The SII is an absolute measure of inequality that considers all population subgroups. A weighted sample of the entire population is ordered from the disadvantaged subgroup (at rank 0) to the privileged subgroup to calculate SII (at rank 1). This rating is weighted to consider the population distribution within each category. The population of each subgroup is then considered in terms of its range and the midpoint of this range in the cumulative population distribution. Using a generalized linear model with a logit link, the health indicator of interest is then regressed on this midpoint value, and the projected values of the health indicator are generated for the two extremes (rank 1 and rank 0). Therefore, the difference between the estimated values at rank 1 ($v_{1}$) and rank 0 ($v_{0}$) (covering the entire distribution) generates the SII value:

$SII= v_{1}-v_{0}$

**Measuring inequality for** **equity strata with two categories**

For equity strata with two categories (like place of residence, media exposure, women’s autonomy, mode of delivery, facility delivery), RD and RR were calculated as following:

$RD= R_{high}-R_{low}$,

$RR= \frac{R_{high}}{R_{low}}$,

where, $R_{high}$ is the rate of PNC use of the reference group (like urban/ cesarean delivery/ facility delivery), and $R_{low}$ is the rate of PNC use of the non-referenced group.

**Equiplot**

The equiplot is a data visualization tool that enables us to view all the indicators and their level of coverage simultaneously, providing a visual representation of absolute inequality.
