# Supplementary material for: Comparison of inequality in utilization of postnatal care services between Bangladesh and Pakistan: Evidence from the Demographic and Health Survey 2017–2018
Source: BMC Pregnancy Childbirth. 2023 Jun 22;23:461. doi: 10.1186/s12884-023-05778-0 (PMC10286509; doi:10.1186/s12884-023-05778-0)
Supplement: Supplementary file 2 — Supplementary Material 2 [file 12884_2023_5778_MOESM2_ESM.docx]

| **Supplementary Table 1: Variable’s categorization, labeling and coding** | | |
| --- | --- | --- |
| **Outcome Variables** | **Description** | **Categories** |
| PNC Check of Women within 2 Days by a Skilled Provider | PNC Check of Women during the first 2 days after birth from a medically trained provider. In Bangladesh, medically trained/ skilled provider includes qualified doctors, nurses, midwives, or paramedics; family welfare visitors; community skilled birth attendants; and sub-assistant community medical officers. In Pakistan, doctors, nurses, midwives, and lady health visitors are considered medically trained providers, and skilled assistance includes doctors, nurses, midwives, lady health visitors, or community midwives. | Categorized into two labels and coded as: ‘0’ No, ‘1’ Yes |
| PNC Check of Newborn within 2 Days by a Skilled Provider | PNC Check of Newborn during the first 2 days after birth from a medically trained provider. |  |
| Adequate PNC Content of Newborn within 2 days | Adequate PNC content includes performing six signal functions of newborn during the first 2 days after birth. Signal functions like cord examination, temperature measurement, counseling on danger signs, counseling on breastfeeding, observation of breastfeeding and weight measurement. |  |
| **Equity Strata** | **Description** | **Categories** |
| Women’s Age (Years) | Age of the women in years during last live birth | Categorized into three labels and coded as: ‘1’ 15–19, ‘2’ 20–34, ‘3’ 35-49 |
| Women’s Education | Women’s level of education | Categorized into five labels and coded as: ‘0’ No formal education, ‘1’ Primary education not completed, ‘2’ Primary education completed (Grade 5), ‘3’ Junior school completed (Grade 8), ‘4’ Secondary or higher |
| Place of Residence | Place/ Area of Residence | Categorized into two labels and coded as: ‘1’ Urban, ‘2’ Rural |
| Household Head | Women are head of their household | Categorized into two labels and coded as: ‘0’ No, ‘1’ Yes |
| Household Size | Number of members in the household | Categorized into two labels and coded as: ‘1’ 1-5 members, ‘2’ 6 or more members |
| Employment Status | Women’s currently employment/working status | Categorized into two labels and coded as: ‘0’ Not currently employed, ‘1’ Currently employed  Notes: Employment is categorized as currently working (having worked in the past 7 days, including women who did not work in the past 7 days but who are regularly employed and were absent from work for leave, illness, vacation, or any other such reason) and not currently working. |
| Wealth Quintile | DHS calculated the wealth index (WI) using data from the DHS databases on household asset ownership, household characteristics, household source of drinking water, and household sanitary facilities [26]. The principal components analysis (PCA) technique is used to construct the WI and assigns a score to each household based on chosen household assets. We used the constructed WI in the DHS to divide the population into wealth quintiles. | Categorized into five labels and coded as: ‘1’ Poorest, ‘2’ Poorer, ‘3’ Middle, ‘4’ Richer, ‘5’ Richest |
| Husband’s Education | Husband’s level of education | Categorized into five labels and coded as: ‘0’ No formal education, ‘1’ Primary education not completed, ‘2’ Primary education completed (Grade 5), ‘3’ Junior school completed (Grade 8), ‘4’ Secondary or higher |
| Wanted Last Child | The last live birth was wanted/desired | Categorized into two labels and coded as: ‘0’ No, ‘1’ Yes |
| Last Live Birth Order | Order of the last live birth of the child | Categorized into four labels and coded as: ‘1’ First, ‘2’ Second, ‘3’ Third, ‘4’ Fourth or Higher |
| Sex of last birth | The sex of the child who born recently | Categorized into two labels and coded as: ‘1’ Male, ‘2’ Female |
| Pregnancy Termination History | There was any pregnancy termination incidence | Categorized into two labels and coded as: ‘0’ No, ‘1’ yes |
| Media exposure | Women who were exposure to either radio, television newspaper or all three media at least once a week are considered regularly exposed to that form of media. | Categorized into two labels and coded as: ‘0’ No, ‘1’ yes |
| Women’s autonomy | Women were considered to ‘have autonomy’ if they made decisions alone or jointly with their husbands regarding their own health care, made major household purchases and were able to visit family or relatives. | Categorized into two labels and coded as: ‘0’ No, ‘1’ yes |
| Facility delivery | Delivery in a health facility like public sector, private sector or nongovernment organization facilities. | Categorized into two labels and coded as: ‘0’ No, ‘1’ yes |
| Mode of delivery | The way of delivery | Categorized into two labels and coded as: ‘1’ Cesarean section, ‘2’ Normal delivery |
| Number of ANC visits | How many times visited antenatal care | Categorized into three labels and coded as: ‘0’ Zero, ‘1’ 1 to 4, ‘2’ More than four |

| **Supplementary Table 2:** **Equity strata associated with PNC service indicators in Bangladesh** | | | | | | | | | | | | |
| --- | --- | --- | --- | --- | --- | --- | --- | --- | --- | --- | --- | --- |
| **Equity Strata** | **PNC Check of Women within 2 Days by Skilled Provider** | | | | **PNC Check of Newborns within 2 Days by Skilled Provider** | | | | **Adequate PNC Content of Newborns within 2 days** | | | |
|  | Simple logistic regression | | Multiple logistic regression | | Simple logistic regression | | Multiple logistic regression | | Simple logistic regression | | Multiple logistic regression | |
|  | OR [95% CI] | *P*-value | aOR [95% CI] | *P*-value | OR [95% CI] | *P*-value | aOR [95% CI] | *P*-value | OR [95% CI] | *P*-value | aOR [95% CI] | *P*-value |
| **Women’s Age** |  |  |  |  |  |  |  |  |  |  |  |  |
| 15-19 | 1 |  | - |  | 1 |  | 1 |  | 1 |  | 1 |  |
| 20-34 | 0.92 [0.77-1.09] | 0.32 | - | - | 0.91 [0.76-1.08] | 0.26 | 0.93 [0.55-1.57] | 0.80 | 1.32 [1.01-1.74] | 0.04 | 1.38 [1.00-1.92] | **0.05** |
| 35-49 | 0.76 [0.56-1.03] | 0.09 | - | - | 0.73 [0.54-1.00] | 0.05 | 1.18 [0.44-3.19] | 0.74 | 0.84 [0.51-1.38] | 0.50 | 1.06 [0.57-1.96] | 0.85 |
| **Women’s Education** |  |  |  |  |  |  |  |  |  |  |  |  |
| No formal education | 1 |  | 1 |  | 1 |  | 1 |  | 1 |  | 1 |  |
| Primary education not completed | 1.31 [0.91-1.88] | 0.14 | 1.11 [0.48-2.57] | 0.82 | 1.23 [0.85-1.76] | 0.27 | 0.82 [0.37-1.84] | 0.64 | 1.69 [0.84-3.41] | 0.14 | 1.49 [0.72-3.10] | 0.28 |
| Primary education completed | 1.49 [1.01-2.19] | 0.04 | 0.89 [0.32-2.45] | 0.82 | 1.65 [1.12-2.41] | 0.01 | 1.43 [0.58-3.54] | 0.44 | 2.00 [0.97-4.13] | 0.06 | 1.60 [0.75-3.43] | 0.23 |
| Junior school completed | 3.58 [2.57-4.97] | 0.00 | 1.84 [0.79-4.28] | 0.16 | 3.46 [2.49-4.81] | 0.00 | 1.63 [0.72-3.72] | 0.24 | 2.94 [1.54-5.64] | 0.00 | 1.47 [0.72-2.99] | 0.29 |
| Secondary or higher | 11.69 [8.21-16.64] | 0.00 | 1.32 [0.52-3.39] | 0.56 | 11.46 [8.05-16.30] | 0.00 | 1.52 [0.60-3.86] | 0.38 | 6.95 [3.62-13.34] | 0.00 | 1.92 [0.91-4.05] | 0.09 |
| **Place of Residence** |  |  |  |  |  |  |  |  |  |  |  |  |
| Rural | 1 |  | 1 |  | 1 |  | 1 |  | 1 |  | 1 |  |
| Urban | 2.38 [2.05-2.75] | 0.00 | 1.56 [1.06-2.29] | **0.02** | 2.30 [1.99-2.66] | 0.00 | 1.45 [0.99-2.12] | **0.05** | 1.69 [1.39-2.05] | 0.00 | 1.05 [0.83-1.33] | 0.68 |
| **Woman as Household Head** |  |  |  |  |  |  |  |  |  |  |  |  |
| No | 1 |  | - |  | 1 |  | - |  | 1 |  | - |  |
| Yes | 1.07 [0.87-1.30] | 0.53 | - | - | 1.14 [0.93-1.39] | 0.20 | - | - | 0.98 [0.73-1.31] | 0.88 | - | - |
| **Household Size** |  |  |  |  |  |  |  |  |  |  |  |  |
| 6 or more members | 1 |  | - |  | 1 |  | - |  | 1 |  | - |  |
| 1-5 members | 1.02 [0.90-1.17] | 0.72 | - | - | 1.01 [0.88-1.15] | 0.92 | - | - | 1.10 [0.91-1.34] | 0.33 | - | - |
| **Employment Status** |  |  |  |  |  |  |  |  |  |  |  |  |
| Not currently employed | 1 |  | 1 |  | 1 |  | 1 |  | 1 |  | 1 |  |
| Currently employed | 0.56 [0.49-0.65] | 0.00 | 1.25 [0.91-1.72] | 0.17 | 0.58 [0.50-0.66] | 0.00 | 1.32 [0.97-1.79] | 0.0.08 | 0.75 [0.61-0.91] | 0.00 | 1.10 [0.87-1.39] | 0.44 |
| **Wealth Quintile** |  |  |  |  |  |  |  |  |  |  |  |  |
| Poorest | 1 |  | 1 |  | 1 |  | 1 |  | 1 |  | 1 |  |
| Poorer | 1.75 [1.41-2.18] | 0.00 | 1.47 [0.97-2.21] | 0.07 | 1.84 [1.48-2.29] | 0.00 | 1.71 [1.13-2.59] | **0.01** | 1.31 [0.91-1.90] | 0.15 | 0.88 [0.58-1.35] | 0.56 |
| Middle | 3.10 [2.48-3.87] | 0.00 | 1.90 [1.18-3.06] | **0.01** | 3.24 [2.60-4.05] | 0.00 | 2.12 [1.36-3.29] | **0.00** | 1.65 [1.15-2.37] | 0.01 | 0.72 [0.46-1.12] | 0.15 |
| Richer | 4.52 [3.62-5.64] | 0.00 | 1.58 [0.92-2.72] | 0.10 | 4.68 [3.75-5.84] | 0.00 | 1.74 [1.08-2.82] | **0.02** | 2.16 [1.52-3.05] | 0.00 | 0.72 [0.46-1.14] | 0.16 |
| Richest | 14.65 [11.39-18.84] | 0.00 | 4.61 [2.58-8.23] | **0.00** | 13.63 [10.63-17.48] | 0.00 | 3.16 [1.66-6.02] | **0.00** | 4.36 [3.17-6.02] | 0.00 | 0.92 [0.57-1.49] | **0.04** |
| **Husband’s Education** |  |  |  |  |  |  |  |  |  |  |  |  |
| No formal education | 1 |  | 1 |  | 1 |  | 1 |  | 1 |  | 1 |  |
| Primary education not completed | 1.31 [1.02-1.69] | 0.03 | 1.07 [0.60-1.91] | 0.81 | 1.30 [1.01-1.67] | 0.04 | 1.00 [0.60-1.67] | 0.99 | 1.11 [0.73-1.70] | 0.62 | 0.86 [0.54-1.39] | 0.55 |
| Primary education completed | 1.81 [1.39-2.36] | 0.00 | 1.06 [0.55-2.04] | 0.85 | 1.78 [1.37-2.31] | 0.00 | 0.96 [0.52-1.78] | 0.89 | 1.45 [0.95-2.22] | 0.09 | 0.84 [0.53-1.36] | 0.48 |
| Junior school completed | 3.01 [2.39-3.80] | 0.00 | 1.19 [0.67-2.11] | 0.55 | 2.98 [2.36-3.75] | 0.00 | 1.11 [0.64-1.91] | 0.71 | 1.88 [1.30-2.72] | 0.00 | 0.83 [0.53-1.30] | 0.41 |
| Secondary or higher | 9.00 [7.00-11.57] | 0.00 | 1.89 [0.99-3.59] | 0.07 | 8.44 [6.58-10.82] | 0.00 | 1.43 [0.76-2.68] | 0.27 | 3.69 [2.58-5.29] | 0.00 | 0.85 [0.52-1.40] | 0.53 |
| **Wanted Last Child** |  |  |  |  |  |  |  |  |  |  |  |  |
| No | 1 |  | 1 |  | 1 |  | 1 |  | 1 |  | 1 |  |
| Yes | 1.58 [1.34-1.86] | 0.00 | 0.96 [0.64-1.43] | 0.84 | 1.60 [1.36-1.88] | 0.00 | 1.05 [0.71-1.54] | 0.82 | 1.74 [1.34-2.26] | 0.00 | 1.25 [0.94-1.66] | 0.13 |
| **Last Live Birth Order** |  |  |  |  |  |  |  |  |  |  |  |  |
| Fourth or higher | 1 |  | 1 |  | 1 |  | 1 |  | 1 |  | 1 |  |
| First | 4.68 [3.69-5.95] | 0.00 | 1.23 [0.67-2.27] | 0.51 | 4.76 [3.75-6.05] | 0.00 | 1.39 [0.72-2.67] | 0.33 | 3.21 [2.12-4.90] | 0.00 | 1.09 [0.66-1.82] | 0.73 |
| Second | 2.72 [2.14-3.47] | 0.00 | 0.83 [0.46-1.47] | 0.52 | 2.72 [1.13-3.46] | 0.00 | 0.89 [0.51-1.57] | 0.69 | 2.64 [1.72-4.07] | 0.00 | 1.10 [0.67-1.80] | 0.70 |
| Third | 1.99 [1.52-2.60] | 0.00 | 1.09 [0.59-2.01] | 0.78 | 2.07 [1.59-2.71] | 0.00 | 1.42 [0.78-2.55] | 0.25 | 1.52 [0.94-2.47] | 0.09 | 0.81 [0.47-1.38] | 0.43 |
| **Sex of Last Birth** |  |  |  |  |  |  |  |  |  |  |  |  |
| Female | 1 |  | - |  | 1 |  | - |  | 1 |  | 1 |  |
| Male | 1.06 [0.93-1.20] | 0.41 | - | - | 1.09 [0.96-1.24] | 0.20 | - | - | 1.30 [1.07-1.57] | 0.01 | 1.25 [1.01-1.54] | 0.07 |
| **Pregnancy Termination History** |  |  |  |  |  |  |  |  |  |  |  |  |
| No | 1 |  | - |  | 1 |  | - |  | 1 |  | - |  |
| Yes | 1.15 [0.96-1.37] | 0.13 | - | - | 1.07 [0.90-1.27] | 0.46 | - | - | 0.97 [0.75-1.27] | 0.85 | - | - |
| **Media exposure** |  |  |  |  |  |  |  |  |  |  |  |  |
| No | 1 |  | 1 |  | 1 |  | 1 |  | 1 |  | 1 |  |
| Yes | 2.96 [2.58-3.39] | 0.00 | 0.77 [0.54-1.10] | 0.15 | 3.03 [2.64-3.47] | 0.00 | 1.02 [0.73-1.42] | 0.90 | 2.35 [1.90-2.90] | 0.00 | 1.21 [0.92-1.58] | 0.17 |
| **Women’s autonomy** |  |  |  |  |  |  |  |  |  |  |  |  |
| No | 1 |  | - |  | 1 |  | - |  | 1 |  | - |  |
| Yes | 1.01 [0.89-1.15] | 0.89 | - | - | 0.98 [0.86-1.12] | 0.78 | - | - | 1.19 [0.98-1.44] | 0.09 | - | - |
| **Facility delivery** |  |  |  |  |  |  |  |  |  |  |  |  |
| No | 1 |  | 1 |  | 1 |  | 1 |  | 1 |  | 1 |  |
| Yes | 598.83 [405.63-884.07] | 0.00 | 299.20 [182.58-490-32] | **0.00** | 516.29 [359.96-740-50] | 0.00 | 295.93 [178.30-491.17] | **0.00** | 14.83 [10.69-20.58] | 0.00 | 8.89 [5.99-13.17] | **0.00** |
| **Mode of Delivery** |  |  |  |  |  |  |  |  |  |  |  |  |
| Normal delivery | 1 |  | 1 |  | 1 |  | 1 |  | 1 |  | 1 |  |
| Cesarean section | 240.37 [132.45-436.22] | 0.00 | 2.86 [1.35-6.07] | **0.01** | 168.78 [106.54-267.37] | 0.00 | 1.83 [0.95-3.53] | 0.07 | 6.31 [5.12-7.77] | 0.00 | 1.49 [1.16-1.92] | **0.00** |
| **Number of ANC Visits** |  |  |  |  |  |  |  |  |  |  |  |  |
| Zero | 1 |  | 1 |  | 1 |  | 1 |  | 1 |  | 1 |  |
| 1 to 4 | 5.02 [3.48-7.24] | 0.00 | 1.23 [0.68-2.21] | 0.49 | 4.74 [3.31-6.79] | 0.00 | 1.08 [0.59-2.00] | 0.80 | 7.62 [2.40-24.15] | 0.00 | 2.93 [0.91-9.47] | 0.07 |
| More than four | 14.80 [10.18-21.51] | 0.00 | 1.43 [0.76-2.67] | 0.27 | 14.09 [9.76-20.34] | 0.00 | 1.37 [0.72-2.61] | 0.35 | 15.92 [5.03-50.45] | 0.00 | 3.28 [1.01-10.66] | **0.05** |
| OR: Odds ratio; aOR: Adjusted odds ratio; CI: Confidence interval; Bold values are statistically significant *p-*values at 5% level. | | | | | | | | | | | | |

| **Supplementary Table 3:** **Equity strata associated with PNC service indicators in Pakistan** | | | | | | | | | | | | |
| --- | --- | --- | --- | --- | --- | --- | --- | --- | --- | --- | --- | --- |
| **Equity Strata** | **PNC Check of Women within 2 Days by Skilled Provider** | | | | **PNC Check of Newborns within 2 Days by Skilled Provider** | | | | **Adequate PNC Content of Newborns within 2 days** | | | |
|  | Simple logistic regression | | Multiple logistic regression | | Simple logistic regression | | Multiple logistic regression | | Simple logistic regression | | Multiple logistic regression | |
|  | OR [95% CI] | *P*-value | aOR [95% CI] | *P*-value | OR [95% CI] | *P*-value | aOR [95% CI] | *P*-value | OR [95% CI] | *P*-value | aOR  [95% CI] | *P*-value |
| **Women’s Age** |  |  |  |  |  |  |  |  |  |  |  |  |
| 15-19 | 1 |  | 1 |  | 1 |  | - |  | 1 |  | 1 |  |
| 20-34 | 1.46 [1.01-2.12] | 0.04 | 1.20 [0.65-2.23] | 0.52 | 1.26 [0.87-1.81] | 0.23 | - | - | 4.78 [1.60-14.26] | 0.01 | 3.02 [0.83-10.95] | 0.09 |
| 35-49 | 1.11 [0.74-1.69] | 0.61 | 1.73 [0.85-3.51] | 0.60 | 0.90 [0.60-1.36] | 0.63 | - | - | 5.27 [1.67-16.68] | 0.01 | 5.34 [1.32-21.71] | **0.02** |
| **Women’s Education** |  |  |  |  |  |  |  |  |  |  |  |  |
| No formal education | 1 |  | 1 |  | 1 |  | 1 |  | 1 |  | 1 |  |
| Primary education not completed | 1.52 [1.01-2.28] | 0.04 | 1.09 [0.60-1.98] | 0.77 | 1.73 [1.15-2.60] | 0.01 | 1.40 [0.73-2.70] | 0.31 | 1.21 [0.43-3.42] | 0.72 | 0.70 [0.23-2.09] | 0.52 |
| Primary education completed | 2.15 [1.57-2.96] | 0.00 | 1.24 [0.76-2.02] | 0.39 | 1.73 [1.26-2.37] | 0.00 | 0.78 [0.48-1.26] | 0.31 | 3.34 [1.76-6.34] | 0.00 | 1.52 [0.73-3.13] | 0.26 |
| Junior school completed | 3.51 [2.55-4.84] | 0.00 | 1.70 [1.00-2.91] | **0.05** | 2.93 [2.13-4.03] | 0.00 | 1.17 [0.68-1.99] | 0.58 | 6.58 [3.67-11.79] | 0.00 | 2.03 [1.03-3.99] | **0.04** |
| Secondary or higher | 7.44 [5.75-9.62] | 0.00 | 1.36 [0.84-2.24] | 0.21 | 6.89 [5.29-8.99] | 0.00 | 1.35 [0.84-2.17] | 0.21 | 10.89 [6.96-17.04] | 0.00 | 1.99 [1.09-3.64] | **0.03** |
| **Place of Residence** |  |  |  |  |  |  |  |  |  |  |  |  |
| Rural | 1 |  | 1 |  | 1 |  | 1 |  | 1 |  | 1 |  |
| Urban | 2.90 [2.40-3.50] | 0.00 | 1.13 [0.83-1.54] | 0.44 | 2.83 [2.34-3.43] | 0.00 | 1.17 [0.82-1.66] | 0.38 | 4.46 [3.16-6.28] | 0.00 | 1.49 [0.98-2.27] | 0.06 |
| **Woman as Household Head** |  |  |  |  |  |  |  |  |  |  |  |  |
| No | 1 |  | - |  | 1 |  | - |  | 1 |  | - |  |
| Yes | 0.94 [0.70-1.26] | 0.69 | - | - | 0.96 [0.71-1.28] | 0.77 | - | - | 0.99 [0.56-1.73] | 0.97 | - | - |
| **Household Size** |  |  |  |  |  |  |  |  |  |  |  |  |
| 6 or more members | 1 |  | - |  | 1 |  | - |  | 1 |  | - |  |
| 1-5 members | 1.07 [0.86-1.33] | 0.54 | - | - | 1.04 [0.83-1.29] | 0.77 | - | - | 1.38 [0.95-2.01] | 0.10 | - | - |
| **Employment Status** |  |  |  |  |  |  |  |  |  |  |  |  |
| Not currently employed | 1 |  | - |  | 1 |  | - |  | 1 |  | - |  |
| Currently employed | 0.94 [0.73-1.22] | 0.67 | - | - | 0.96 [0.74-1.24] | 0.73 | - | - | 1.13 [0.71-1.82] | 0.60 | - | - |
| **Wealth Quintile** |  |  |  |  |  |  |  |  |  |  |  |  |
| Poorest | 1 |  | 1 |  | 1 |  | 1 |  | 1 |  | 1 |  |
| Poorer | 0.98 [0.75-1.28] | 0.88 | 0.46 [0.31-0.69] | **0.00** | 1.01 [0.78-1.31] | 0.93 | 0.52 [0.35-0.76] | **0.00** | 2.50 [1.06-5.85] | 0.04 | 1.67 [0.68-4.13] | 0.26 |
| Middle | 1.61 [1.23-2.11] | 0.00 | 0.36 [0.24-0.56] | **0.00** | 1.92 [1.48-2.51] | 0.00 | 0.56 [0.37-0.86] | **0.01** | 5.66 [2.56-12.52] | 0.00 | 2.06 [0.79-5.39] | 0.14 |
| Richer | 3.86 [2.90-5.13] | 0.00 | 0.52 [0.31-0.87] | **0.01** | 3.81 [2.86-5.07] | 0.00 | 0.65 [0.39-1.07] | 0.09 | 12.19 [5.73-25.92] | 0.00 | 2.22 [0.80-6.15] | 0.13 |
| Richest | 9.76 [7.05-13.51] | 0.00 | 0.60 [0.33-1.09] | 0.09 | 8.66 [6.22-12.05] | 0.00 | 0.61 [0.34-1.08] | 0.09 | 28.92 [14.21-58.85] | 0.00 | 3.56 [1.21-10.48] | **0.02** |
| **Husband’s Education** |  |  |  |  |  |  |  |  |  |  |  |  |
| No formal education | 1 |  | 1 |  | 1 |  | 1 |  | 1 |  | 1 |  |
| Primary education not completed | 1.21 [0.78-1.87] | 0.40 | 0.76 [0.43-1.34] | 0.34 | 1.46 [0.95-2.24] | 0.08 | 1.02 [0.54-1.92] | 0.95 | 1.15 [0.31-4.25] | 0.83 | 0.60 [0.15-2.39] | 0.47 |
| Primary education completed | 1.25 [0.90-1.72] | 0.18 | 1.07 [0.67-1.72] | 0.77 | 1.34 [0.97-1.85] | 0.07 | 1.14 [0.73-1.78] | 0.55 | 1.14 [0.50-2.62] | 0.75 | 0.66 [0.27-1.60] | 0.35 |
| Junior school completed | 1.96 [1.49-2.59] | 0.00 | 1.19 [0.76-1.87] | 0.45 | 2.06 [1.56-2.71] | 0.00 | 1.41 [0.89-2.23] | 0.14 | 3.13 [1.67-5.85] | 0.00 | 1.15 [0.56-2.35] | 0.70 |
| Secondary or higher | 4.30 [3.46-5.34] | 0.00 | 1.32 [0.89-1.95] | 0.17 | 4.17 [3.35-5.19] | 0.00 | 1.41 [0.98-2.03] | 0.07 | 5.28 [3.22-8.67] | 0.00 | 0.83 [0.43-1.59] | 0.57 |
| **Wanted Last Child** |  |  |  |  |  |  |  |  |  |  |  |  |
| No | 1 |  | - |  | 1 |  | - |  | 1 |  | - |  |
| Yes | 1.14 [0.86-1.49] | 0.36 | - | - | 1.11 [0.85-1.46] | 0.77 | - | - | 1.09 [0.63-1.87] | 0.77 | - | - |
| **Last Live Birth Order** |  |  |  |  |  |  |  |  |  |  |  |  |
| Fourth or higher | 1 |  | 1 |  | 1 |  | 1 |  | 1 |  | 1 |  |
| First | 2.43 [1.91-3.09] | 0.00 | 1.06 [0.71-1.60] | 0.77 | 2.52 [1.98-3.22] | 0.00 | 1.10 [0.75-1.59] | 0.63 | 1.76 [1.14-2.73] | 0.01 | 0.86 [0.50-1.48] | 0.58 |
| Second | 1.93 [1.53-2.45] | 0.00 | 1.20 [0.81-1.79] | 0.36 | 1.80 [1.42-2.27] | 0.00 | 1.03 [0.71-1.50] | 0.88 | 1.58 [1.00-2.50] | 0.05 | 0.83 [0.49-1.41] | 0.49 |
| Third | 1.65 [1.28-2.13] | 0.00 | 1.25 [0.84-1.86] | 0.27 | 1.52 [1.18-1.95] | 0.00 | 1.03 [0.72-1.47] | 0.89 | 1.55 [0.96-2.51] | 0.07 | 0.93 [0.53-1.64] | 0.81 |
| **Sex of Last Birth** |  |  |  |  |  |  |  |  |  |  |  |  |
| Female | 1 |  | - |  | 1 |  | - |  | 1 |  | - |  |
| Male | 0.94 [0.79-1.12] | 0.48 | - | - | 0.91 [0.77-1.09] | 0.32 | - | - | 0.80 [0.58-1.11] | 0.19 | - | - |
| **Pregnancy Termination History** |  |  |  |  |  |  |  |  |  |  |  |  |
| No | 1 |  | - |  | 1 |  | - |  | 1 |  | - |  |
| Yes | 0.97 [0.80-1.18] | 0.77 | - | - | 0.97 [0.80-1.18] | 0.74 | - | - | 0.91 [0.63-1.31] | 0.61 | - | - |
| **Media exposure** |  |  |  |  |  |  |  |  |  |  |  |  |
| No | 1 |  | 1 |  | 1 |  | 1 |  | 1 |  | 1 |  |
| Yes | 2.60 [2.17-3.11] | 0.00 | 1.47 [1.08-2.01] | **0.01** | 2.55 [2.13-3.06] | 0.00 | 1.51 [1.11-2.05] | **0.01** | 4.31 [2.90-6.42] | 0.00 | 1.51 [0.94-2.42] | 0.09 |
| **Women’s autonomy** |  |  |  |  |  |  |  |  |  |  |  |  |
| No | 1 |  | 1 |  | 1 |  | 1 |  | 1 |  | 1 |  |
| Yes | 1.95 [1.60-2.37] | 0.00 | 1.65 [1.20-2.26] | **0.00** | 2.12 [1.73-2.59] | 0.00 | 2.21 [1.61-3.04] | **0.00** | 1.94 [1.39-2.71] | 0.00 | 1.34 [0.93-1.94] | 0.12 |
| **Facility delivery** |  |  |  |  |  |  |  |  |  |  |  |  |
| No | 1 |  | 1 |  | 1 |  | 1 |  | 1 |  | 1 |  |
| Yes | 82.89 [56.37-121.88] | 0.00 | 47.21 [31.71-71.30] | **0.00** | 67.18 [47.22-95.57] | 0.00 | 44.42 [30.50-64.70] | **0.00** | 31.94 [12.52-81.48] | 0.00 | 10.55 [3.97-28.02] | **0.00** |
| **Mode of Delivery** |  |  |  |  |  |  |  |  |  |  |  |  |
| Normal delivery | 1 |  | 1 |  | 1 |  | 1 |  | 1 |  | 1 |  |
| Cesarean section | 31.10 [19.43-49.78] | 0.00 | 5.57 [3.39-9.15] | **0.00** | 14.03 [9.43-20.87] | 0.00 | 2.08 [1.35-3.21] | **0.00** | 5.87 [4.18-8.26] | 0.00 | 1.84 [1.23-2.75] | **0.00** |
| **Number of ANC Visits** |  |  |  |  |  |  |  |  |  |  |  |  |
| Zero | 1 |  | 1 |  | 1 |  | 1 |  | 1 |  | 1 |  |
| 1 to 4 | 5.98 [4.24-8.43] | 0.00 | 3.09 [2.04-4.68] | **0.00** | 5.43 [3.96-7.44] | 0.00 | 2.64 [1.80-3.86] | **0.00** | 16.00 [3.62-70.69] | 0.00 | 4.88 [1.04-22.94] | **0.05** |
| More than four | 30.87 [21.34-44.66] | 0.00 | 6.24 [3.90-9.99] | **0.00** | 24.71 [17.47-34.95] | 0.00 | 4.62 [3.02-7.05] | **0.00** | 63.35 [14.56-275.65] | 0.00 | 6.39 [1.36-29.93] | **0.02** |
| OR: Odds ratio; aOR: Adjusted odds ratio; CI: Confidence interval; Bold values are statistically significant *p-*values at 5% level. | | | | | | | | | | | | |
